# Triangular Mechanical Structure of the Proximal Femur

**DOI:** 10.1111/os.13498

**Published:** 2022-09-30

**Authors:** Gaoxiang Xu, Jiantao Li, Cheng Xu, Dou Xiong, Hua Li, Daofeng Wang, Wupeng Zhang, Hao Zhang, Licheng Zhang, Peifu Tang

**Affiliations:** ^1^ Department of Orthopaedics Chinese PLA General Hospital Beijing China; ^2^ National Clinical Research Center for Orthopaedics Sports Medicine & Rehabilitaion Beijing China

**Keywords:** anatomic measurement, computed tomography, finite element analysis, proximal femur, triangular mechanical structure

## Abstract

**Objective:**

The mechanical high modulus structure of the proximal femur could guide clinical surgical treatment and instrument design of proximal femoral fractures. The purpose of this study is to analyze and verify the mechanical structure of the proximal femur.

**Methods:**

A total of 375 patients with intertrochanteric fractures were imaged using computed tomography (CT) scans. Patients were grouped according to age and sex. Cortical and medullary cavity parameters (cortical thickness [CTh], cortical mean density [CM], upper‐lower diameter length [ULL], and medial‐lateral diameter length [MLL]) were measured at eight planes. Six proximal femoral finite element models of different sexes and ages were constructed. To verify the measurement results, Abaqus was used to implement the force load to describe the von Mises stress distribution, and the maximum von Mises stress values of each wall of the proximal femur were compared.

**Results:**

The CTh values of the lower and upper walls were higher than those of the anterior and posterior walls of the femoral neck (*p* < 0.05). The CM values of the lower and upper walls were higher than those of the anterior and posterior walls of the subcephalic and middle femoral neck (*p* < 0.05). The ULL value gradually increased from the subcephalic region to the bottom (*p* < 0.05). The CTh and CM values of the medial and lateral walls were higher than those of the anterior and posterior walls in the femoral trochanteric region (*p* < 0.05). The MLL value decreased gradually from the plane 20 mm above the upper edge to that 20 mm below the vertex of the femoral lesser trochanter (*p* < 0.05). The von Mises stress was concentrated on the upper and lower walls of the femoral neck and on the medial and lateral walls of the femoral trochanteric region. The maximum von Mises stress values of the upper and lower walls were higher than those of the anterior and posterior walls of the femoral neck. The maximum von Mises stress values of the medial and lateral walls were higher than those of the anterior and posterior walls in the femoral trochanteric region, except for the plane 20 mm above the upper edge of the femoral lesser trochanter.

**Conclusion:**

The bone mass of the proximal femur presented a triangular high‐modulus distribution, which bore the main stress of the proximal femur. The triangular mechanical structure provides a guideline for the surgical strategy and instrument design of the proximal femur.

## Graphical abstract

Results of the anatomic measurements confirmed that cortical bone varied dramatically across cross‐section, with prominent cortical thickening, densifying and triangular convergence at the upper, medial, and lateral walls of the proximal femur. Finite element analysis verified the measurement results. Thus, a triangular mechanical structure exists in the proximal femur.

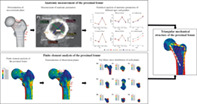

## Introduction

With the increasing age of the population, the number of patients with hip fractures will reach 4.5 million by the year 2050, and proximal femoral fractures account for approximately 90% of hip fractures^1^. Surgery should be performed as soon as possible to avoid complications caused by long‐term bed rest^2^. However, studies have shown that loss of fracture reduction occurs in 40% of patients postoperatively^3^. Thus, ensuring the success of an operation has attracted considerable attention.

The AO (Arbeitsgemeinschaftfür Osteosynthesefragen) principle of fracture management emphasizes that the primary principle of surgical treatment is to realize the mechanical integrity of the fracture site through internal fixation instruments in line with the local mechanical structure^4^. An accurate understanding of the mechanical structure is very important for the treatment of proximal femoral fractures. Through analogy and theoretical analysis, Ward^5^, Culmann^6^, and Koch^7^ regarded the cantilever structure as the standard mechanical structure that was applied to guide the treatment of proximal femoral fractures. Based on this mechanical structure, an extramedullary fixation system, such as a Jewett‐angle steel plate^8^ or sliding screw plate^9^, has been developed and utilized in clinical treatment. However, with a further understanding of the mechanical structure of the proximal femur, it was found that the extramedullary fixation system, as an eccentric design, has a long force arm, resulting in a high stress concentration at the tip of the head nail. The junction between the head nail and the main nail exceeds the yield stress of the bone and fixation system, which is the cause of frequent complications including nail breakage, cut‐out, and varus collapse^10^. Therefore, intramedullary fixation systems, such as gamma nail^11^, Proximal Femoral Nail Antirotation (PFNA)^12^, and InterTan^13^, have been developed to improve the surgical effect. However, most existing intramedullary fixation systems cannot reconstruct the medial support structure, which is important for the treatment of proximal femoral fractures, especially unstable fractures^14^. A postoperative complication rate of up to 20.5% was reported for the existing intramedullary fixation systems^15^.

Therefore, we propose that the characteristics of the mechanical structure of the proximal femur have not been clarified. Through retrospective analysis of 53 failed cases of fracture treatment, we found that the failure of any unilateral reconstruction of the medial, lateral, and upper walls of the proximal femur will lead to the failure of hip fracture fixation^16^. For the treatment of femoral intertrochanteric fractures, we designed a medial sustainable nail with a triangular structure, exhibiting a better biomechanical performance when compared with that of PFNA in reducing displacement and anti‐varus^17^. In addition, for displaced femoral neck fractures, a medial anatomical buttress plate capable of forming a triangular structure was designed with more stable properties with respect to the stress distributions, stress peaks, and Z‐axis displacements, and all healing was achieved in the treatment of 15 patients with femoral neck fracture nonunion^18^, ^19^. Thus, we speculated whether there was a triangular mechanical structure in the proximal femur, so that any strategy realizing complete reconstruction of the triangular structure could be successful.

In this study, the anatomical structural parameters of the proximal femoral cortex bearing 47%–80% of the overall stress^20^, ^21^ were measured in 375 patients with intertrochanteric fractures, and the normal stress distribution of the proximal femur in six patients was analyzed. The aims of this study were: (i) to investigate the high‐modulus structural morphology of bone mass in the proximal femur and (ii) to investigate the mechanical effect of the above structure.

## Methods

### Patients

From September 2009 to March 2017, 375 patients with intertrochanteric fractures who underwent surgery at the Chinese PLA General Hospital were included in our study to assess cortical structural characteristics of the proximal femurs. This study was approved by the institutional review board of our hospital (S2020‐114‐04).

Inclusion criteria were patients (1) older than 40 years, (2) with low‐energy external injuries such as a simple fall from standing height or lower and twisting injuries^22^, (3) with CT scanning range from the upper edge of the first lumbar vertebra to 8 cm below the lesser trochanter of the femur, and (4) with imaging follow‐up for at least 1 year. The exclusion criteria were as follows: (1) femoral head necrosis, (2) severe hip osteoarthritis or rheumatoid arthritis, (3) hip joint or femur deformity, and (4) history of contralateral hip fracture.

All CT data were collected using one CT machine (Siemens AG, Erlangen, Germany) with the same scanning parameters (120 KV, 210 mA; collimation, 4 mm; table speed, 3–5 mm/s; slice thickness 1.2 mm with 1.5 mm interval). In Mimics 20.0 (Materialize Inc., Leuven, Belgium), the osseous material was isolated *via* thresholding at 0–350 Hounsfield units, which could clearly display the outline of bone.

Patients were divided into two groups according to sex: Group G1 (male) and Group G2 (female). The patients were then classified into three groups according to age: Group A1 (40–59 years), Group A2 (60–79 years), Group A3 (≥80 years).

### Anatomical Measurement of the Proximal Femur

**Fig. 1:**
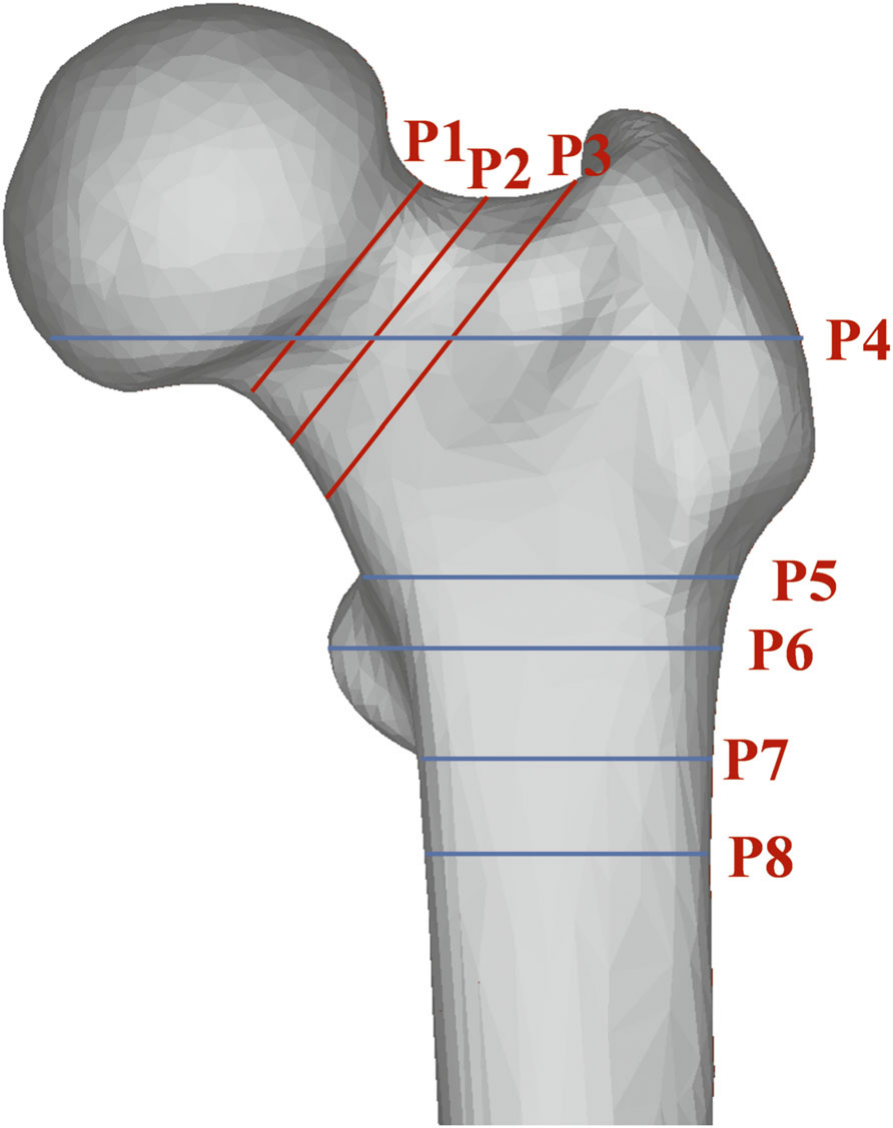
Eight planes at different sites of the proximal femur. The femoral neck was divided by axial P1–3 (planes at the subcephalic, middle, and bottom sites of the femoral neck, respectively). Similarly, the trochanteric region was divided by P4–8 (P4, plane at the 20 mm above the upper edge of femoral lesser trochanter; P5, plane at the upper edge of femoral lesser trochanter; P6, plane at the vertex of femoral lesser trochanter; P7, plane at the lower edge of femoral lesser trochanter; P8, plane at the 20 mm below the vertex of femoral lesser trochanter)

**Fig. 2:**
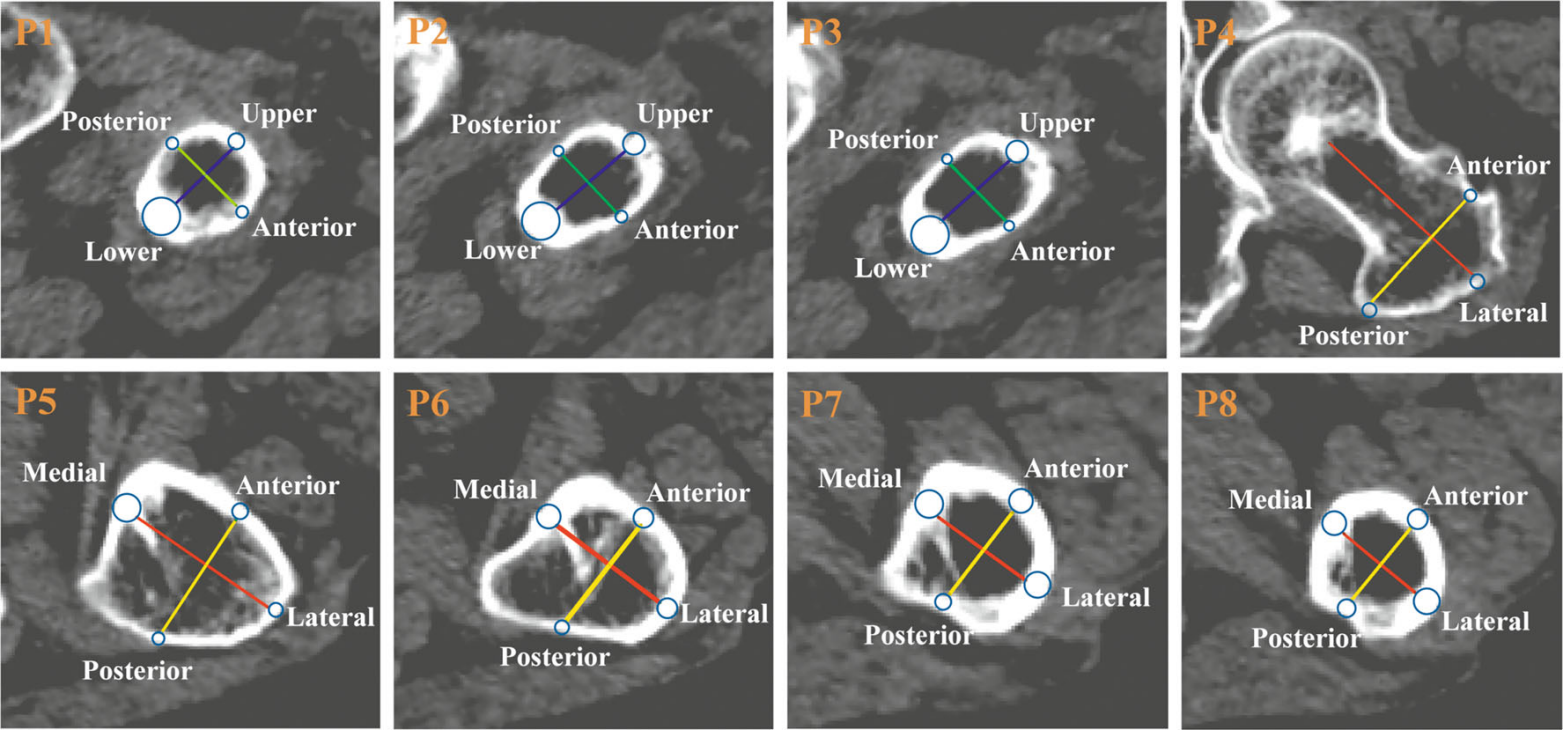
Locations of the measurement points and lines in the proximal femoral region. (P1–3): Femoral neck region. Blue line indicated the longest diameter between upper and lower walls. Green line indicated the longest diameter between anterior and posterior walls. (P4–8): Femoral trochanteric region. Red line indicated the longest diameter between medial and lateral walls. Yellow line indicated the longest diameter between anterior and posterior walls. Blue circle was the tangent circle in the cortex corresponding to the straight line

In the femoral neck region, cortical bone was defined as the upper, lower, anterior, and posterior walls. The bottom of the femoral neck was determined using the method described by Zhang^23^, the subcephalic part of the femoral neck was determined according to Sparks^24^, and then the middle part of the femoral neck was determined (Figure 1). In planes 1–3, blue lines (the longest line between the upper and lower walls) 112 were drawn with lengths measured as ULL and green lines (the longest line between the anterior and posterior walls) were drawn. The regions of the circle tangent to the cortical bone (where the blue and green lines intersect with the cortical bone) were identified as the measurement regions (Figure 2, P1–3).

In the trochanteric region, the cortical bone was defined by the medial, lateral, anterior, and posterior walls. Using the method described by Zhang^25^, Planes 4–8 were determined (Figure 1). In each plane, the red line (the longest line between the medial and lateral walls) was drawn with the length measured as MLL, and the yellow line (the longest line between the anterior and posterior walls) was drawn. The measurement regions of the medial, anterior, lateral, and posterior walls in planes 4–8 were defined to extract the relevant anatomical parameters (Figure 2, P4–8). The diameter and mean Hounsfield unit values of these regions were regarded as the cortical thickness (CTh) and cortical mean density (CM) values, respectively, of each wall.

### Finite Element Analysis of the Proximal Femur

Finite element analysis was used to describe the stress distribution of the proximal femur in the different age and sex groups. Using the RAND function in Microsoft Excel 2016, the same‐sex patients in the three age groups were sorted by the value received from the random number generation function, and six patients with serial number 1 (Model 1: female, 56 years, 156 cm, 64 kg; Model 2: male, 57 years, 180 cm, 60 kg; Model 3: female, 64 years, 165 cm, 45 kg; Model 4: male, 62 years, 170 cm, 66 kg; Model 5: female, 88 years, 160 cm, 60 kg; Model 6: male, 82 years, 172 cm, 60 kg) were selected from the 375 patients. Their consecutive CT data were imported into Mimics 20.0, and three‐dimensional models of their proximal femurs were constructed through the protocols of thresholding, region growing, mask editing, polyline calculation, cavity filling from polylines, etc. The threshold units ranged from 226 to 3071. The “region growing” tool was utilized to split the segmentation as desired and to remove floating pixels. The polyline calculation was used to edit the details of the proximal femur. After the procedures above, six three‐dimensional reconstruction models of the proximal femur were generated and further imported into Geomagic Studio 14.0 (Geomagic, Inc., Research Triangle Park, NC, USA) in STL format for modification. Using the material function of the FEA module in the Mimics software, the density and elastic modulus of the proximal femoral bone structure were automatically defined according to the CT value of the bone structure^26^, and the Poisson's ratio of all bone structures was defined as 0.3^27^. Finally, these models were imported into Abaqus (Simulia, France) to generate the finite element models. Subsequently, a mesh sensitivity analysis was performed to determine the correct size of the elements with the lowest computational cost^28^. For all the FE models, element sizes (mm) of 0.65–7.00 (with an interval of 0.05) were used. We used the bisection method to perform a difference analysis of the calculated maximum von Mises stress. The formula is as follows:

(1) $$S=\left|\frac{X_{2n}-X_{n}}{X_{2n}}\right|$$

where *S* is the difference ratio, *X* is the maximum von Mises stress value, and *n* is the mesh size. In total, 17 difference ratios were obtained. We performed linear regression analysis on the difference ratio results and obtained the regression curve *Y* = 0.0242*X* + 0.0017, where *Y* represents the difference ratio, and therefore the FE accuracy; *X* represents the mesh size. When *Y* is set as 0.05, we obtain *X* = 2, which means that a 2 mm mesh size guarantees an accurate result; therefore, we set the mesh size to 2 mm. Warpage, aspect ratio, and Jacobian elements were used to assess the mesh quality. In all models used in this study, the element Jacobian was above 0.7, warpage was less than 5, and the aspect ratio was less than 5, which indicates a good mesh quality. Linear tetrahedral elements (C3D4) were applied to the finite element models. All nodes on the surface of the distal femoral shaft were constrained with 0° of freedom to avoid rigid‐body motion during the analysis. This study simulated the force loading on the hip during the stance phase of walking. According to the body weight of the volunteers and the literature^29^, the weight‐bearing forces of the femoral head of models 1–6 were set to 1920N, 1800N, 1350N, 1980N, 1800N, and 1800N respectively, corresponding to 300% body weight. The net force was applied to the femoral head at 9° posteriorly on the sagittal plane and 10° laterally in the coronal plane^30^. The von Mises stress distribution in the proximal femur is also shown. As in the measurement sites of anatomical parameters of each section of the proximal femur, the maximum von Mises stress value of the same region in eight sections of models 1–6 was recorded to reflect the stress in different walls.

### Reliability Study

The intraclass correlation coefficient (ICC) was used to assess the reliability of the measurement methods established in this study. PASS 11.0 (NCSS LLC. Kaysville, UT) was used to estimate the sample size for the intraclass correlation coefficient. The design solved for sample size: A random sample of several subjects who were each measured three times produced a two‐sided 95% confidence interval with a width of 0.1 when the estimated intraclass correlation was 0.950. Data were analyzed using a two‐way random effects ANOVA model. Given these settings, the minimum sample size was calculated to be 14. To increase the reliability, 20 femurs were selected for the reliability study. One independent physician (GXX) repeated the measurements of 70 proximal femoral parameters three times in a randomized order with a minimum of 24‐h intervals to assess intraobserver reliability. Three other physicians (JTL, HZ, and CX) independently measured the same parameters on the same samples in a random order to assess interobserver reliability. Two‐way random and two‐way fixed models were selected to assess inter‐ and intra‐observer reliability.

### Statistical Analysis

All statistical analyses were performed using SPSS 21.0 (IBM, Armonk, NY, USA). The Kolmogorov–Smirnov test was used to detect normally distributed values. The results of the different regions in the proximal femur regarding CTh, CM, ULL, and MLL values within each group were compared using one‐way ANOVA for normally distributed values and Kruskal–Wallis test for non‐normally distributed values. The significance level for all statistical tests was set at *p* < 0.05.

## Results

Group G1 comprised 135 patients, with an average age of 75.39 ± 10.87 years. Group G2 comprised 240 patients, with an average age of 79.18 ± 8.25 years. Group A1 consisted of 16 male and 6 female patients, for a total of 22. Group A2 consisted of 61 male and 110 female patients, totaling 171. Group A3 included 58 male and 124 female patients, totaling 182.

### Anatomical Measurements of the Proximal Femur

**Fig. 3:**
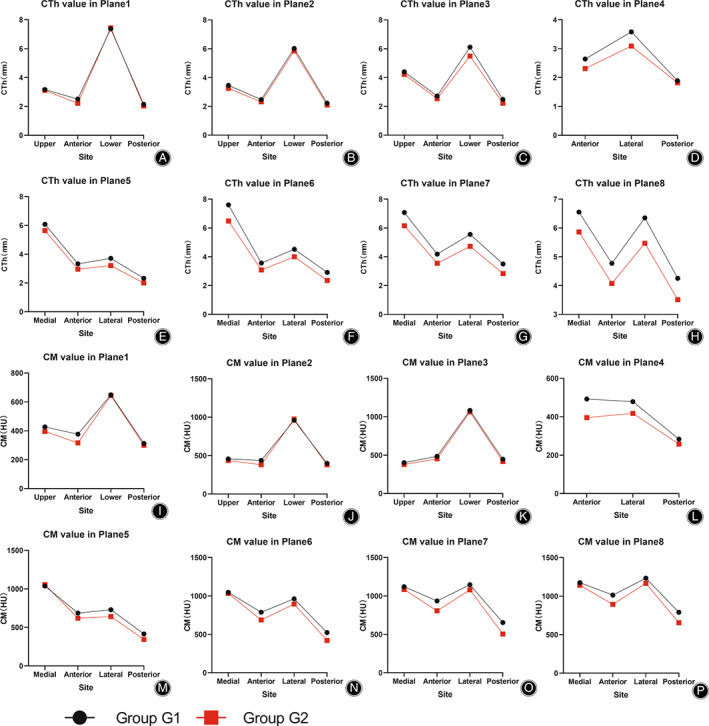
CTh and CM values of each wall in planes 1–8 grouped by sex. (A–H): CTh values of each wall in planes 1–8 grouped by sex; (I–P): CM values of each wall in planes 1–8 grouped by sex

**Fig. 4:**
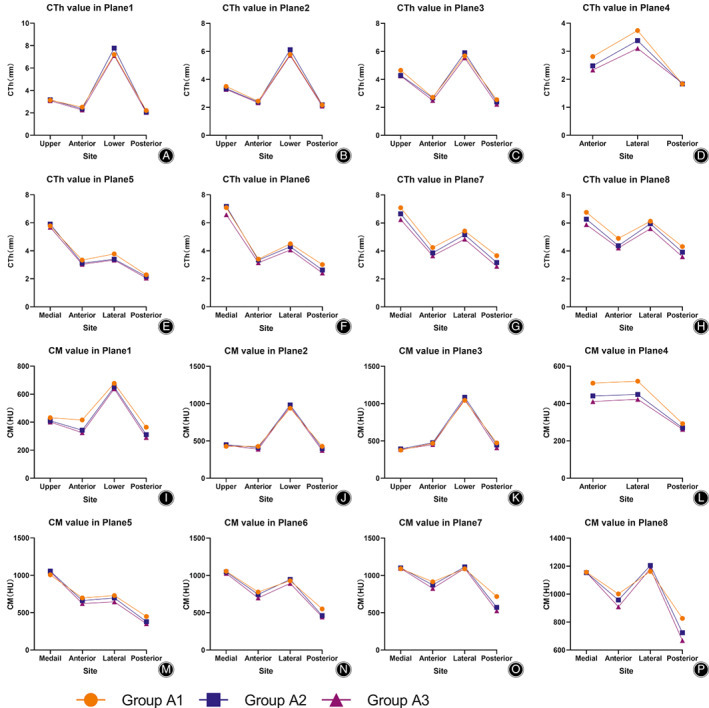
CTh and CM values of each wall in planes 1–8 grouped by age. (A–H): CTh values of each wall in planes 1–8 grouped by age; (I–P): CM values of each wall in planes 1–8 grouped by age

**Fig. 5:**
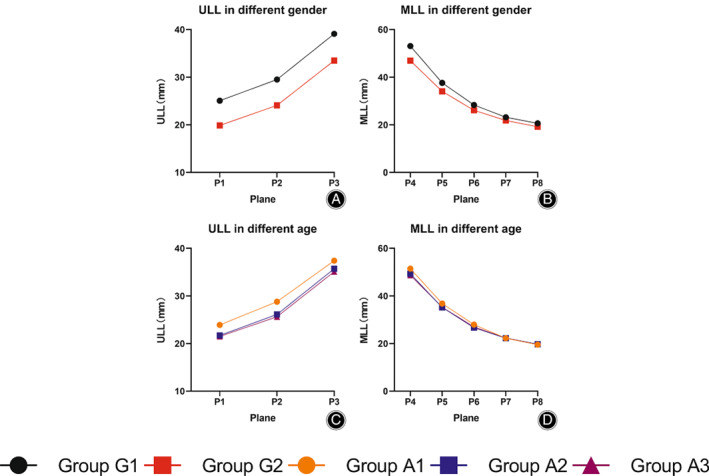
ULL and MLL values grouped by sex and age. (A, B): ULL and MLL in planes 1–8 grouped by sex; (C, D): ULL and MLL in planes 1–8 grouped by age

In the femoral neck region, the CTh values of the lower and upper walls were higher than those of the anterior and posterior walls in planes 1–3, regardless of sex and age (*p* < 0.05) (Figures 3A–C and 4A–C; Tables 1, 2). The CM values of the lower and upper walls were higher than those of the anterior and posterior walls in planes 1–2 regardless of sex and age (*p* < 0.05). The upper wall exhibited the lowest value and the lower wall exhibited the highest value in plane 3 regardless of sex and age (*p* < 0.05) (Figures 3I–K and 4I–K; Tables 1, 2). The ULL values increased gradually from plane 1 to plane 3, regardless of sex and age (*p* < 0.05) (Figure 5A, C; Table 5).

**TABLE 1:** Comparison of CTh and CM values (Mean ± SD, mm, HU) of the femoral neck in different sexes

Site	Group G1		Group G2	
	CTh	CM	CTh	CM
Plane 1				
Upper	3.17 ± 0.80	426.63 ± 110.13	3.11 ± 0.86	395.60 ± 97.73
Anterior	2.51 ± 0.59	376.76 ± 114.83	2.22 ± 0.48	316.72 ± 107.38
Lower	7.37 ± 2.14	648.58 ± 141.94	7.44 ± 1.85	645.11 ± 143.89
Posterior	2.15 ± 0.50	312.75 ± 119.71	2.03 ± 0.43	299.79 ± 95.51
*F*	540.59	192.27	1351.19	479.36
*p*	0.000	0.000	0.000	0.000
Plane 2				
Upper	3.46 ± 0.77	457.89 ± 100.85	3.25 ± 0.640	436.88 ± 96.69
Anterior	2.47 ± 0.54	437.52 ± 112.42	2.31 ± 0.47	381.65 ± 94.91
Lower	6.02 ± 1.39	959.07 ± 164.91	5.85 ± 1.14	975.81 ± 166.55
Posterior	2.22 ± 0.41	399.12 ± 108.92	2.09 ± 0.42	380.15 ± 107.96
*F*	542.51	612.50	1362.67	1391.51
*p*	0.000	0.000	0.000	0.000
Plane 3				
Upper	4.40 ± 1.20	402.32 ± 114.26	4.22 ± 1.22	379.87 ± 127.56
Anterior	2.72 ± 0.69	484.90 ± 115.66	2.53 ± 0.60	452.35 ± 117.28
Lower	6.11 ± 1.36	1082.66 ± 153.73	5.49 ± 1.13	1063.72 ± 154.58
Posterior	2.48 ± 0.54	447.06 ± 105.17	2.22 ± 0.40	417.41 ± 95.97
*F*	379.23	908.77	686.09	1605.56
*p*	0.000	0.000	0.000	0.000

Abbreviations: CM, cortical mean density; CTh, cortical thickness.

**TABLE 2:** Comparison of CTh and CM values (Mean ± SD, mm, HU) of the femoral neck in different ages

Site	Group A1		Group A2		Group A3	
	CTh	CM	CTh	CM	CTh	CM
Plane 1						
Upper	3.14 ± 0.77	431.75 ± 102.43	3.17 ± 0.86	409.70 ± 101.84	3.09 ± 0.83	401.00 ± 104.77
Anterior	2.51 ± 0.57	415.86 ± 115.37	2.37 ± 0.57	342.68 ± 104.53	2.26 ± 0.50	324.88 ± 118.20
Lower	7.21 ± 1.89	677.15 ± 137.05	7.77 ± 2.13	650.83 ± 143.97	7.11 ± 1.75	638.44 ± 142.88
Posterior	2.21 ± 0.56	363.74 ± 134.44	2.07 ± 0.49	310.90 ± 101.97	2.05 ± 0.42	291.23 ± 101.14
*F*	99.30	28.33	828.66	308.71	976.46	321.08
*p*	0.000	0.000	0.000	0.000	0.000	0.000
Plane 2						
Upper	3.50 ± 0.90	428.39 ± 101.04	3.34 ± 0.71	449.78 ± 98.77	3.29 ± 0.65	441.37 ± 98.34
Anterior	2.44 ± 0.62	427.20 ± 104.43	2.39 ± 0.47	411.26 ± 105.54	2.33 ± 0.51	389.77 ± 103.46
Lower	5.78 ± 1.01	938.37 ± 150.48	6.12 ± 1.23	981.38 ± 161.69	5.72 ± 1.24	962.69 ± 171.51
Posterior	2.17 ± 0.38	428.35 ± 117.33	2.18 ± 0.41	396.09 ± 104.21	2.09 ± 0.43	373.43 ± 109.89
*F*	100.15	99.62	940.50	939.98	825.91	936.22
*p*	0.000	0.000	0.000	0.000	0.000	0.000
Plane 3						
Upper	4.65 ± 1.31	377.50 ± 85.09	4.28 ± 1.16	393.12 ± 130.53	4.25 ± 1.25	384.36 ± 120.40
Anterior	2.71 ± 0.75	468.93 ± 128.87	2.68 ± 0.66	476.34 ± 118.95	2.50 ± 0.59	451.95 ± 114.29
Lower	5.68 ± 1.00	1043.77 ± 179.92	5.90 ± 1.26	1085.05 ± 150.59	5.54 ± 1.25	1060.14 ± 154.18
Posterior	2.55 ± 0.51	473.38 ± 129.51	2.38 ± 0.50	444.09 ± 103.52	2.22 ± 0.43	407.57 ± 88.56
*F*	57.86	112.25	500.02	1121.49	484.33	1291.31
*p*	0.000	0.000	0.000	0.000	0.000	0.000

Abbreviations: CM, cortical mean density; CTh, cortical thickness.

In the femoral trochanteric region, the CTh values of the medial and lateral walls were higher than those of the anterior and posterior walls in planes 5–8 regardless of sex and age (*p* < 0.05), and the highest CTh value was found in the lateral wall in plane 4, regardless of sex and age (*p* < 0.05) (Figures 3D–H and 4D–H; Tables 3, 4). The spatial distribution of CM values was similar to that of CTh, regardless of sex and age. (Figures 3L–P and 4L–P; Tables 3, 4). The MLL values decreased gradually from plane 4 to plane 8, regardless of sex and age (*p* < 0.05) (Figure 5B, D; Table 6).

**TABLE 3:** Comparison of CTh and CM values (Mean ± SD, mm, HU) of the femoral trochanteric region in different sexes

Site	Group G1		Group G2	
	CTh	CM	CTh	CM
Plane 4				
Anterior	2.64 ± 0.76	492.22 ± 132.54	2.31 ± 0.58	395.11 ± 118.28
Lateral	3.58 ± 0.95	478.62 ± 128.35	3.09 ± 0.88	417.84 ± 117.60
Posterior	1.89 ± 0.37	283.28 ± 84.01	1.82 ± 0.41	257.70 ± 81.24
*F*	180.01	134.67	234.17	157.02
*p*	0.000	0.000	0.000	0.000
Plane 5				
Medial	6.07 ± 1.59	1037.44 ± 176.20	5.64 ± 1.19	1055.70 ± 158.40
Anterior	3.33 ± 0.81	686.04 ± 136.74	2.96 ± 0.66	619.97 ± 128.79
Lateral	3.71 ± 0.89	730.39 ± 148.11	3.21 ± 0.97	640.58 ± 154.65
Posterior	2.33 ± 0.51	415.42 ± 103.25	2.00 ± 0.45	344.59 ± 90.92
*F*	320.00	425.78	766.58	1117.46
*p*	0.000	0.000	0.000	0.000
Plane 6				
Medial	7.60 ± 2.00	1046.06 ± 171.46	6.48 ± 1.44	1033.41 ± 163.04
Anterior	3.56 ± 0.81	788.55 ± 146.58	3.08 ± 0.69	687.63 ± 156.15
Lateral	4.52 ± 1.02	963.18 ± 170.66	4.00 ± 0.92	894.62 ± 167.98
Posterior	2.91 ± 0.71	523.38 ± 120.70	2.35 ± 0.55	421.17 ± 105.95
*F*	374.95	304.79	838.67	753.66
*p*	0.000	0.000	0.000	0.000
Plane 7				
Medial	7.07 ± 1.75	1120.33 ± 150.24	6.15 ± 1.39	1084.47 ± 162.70
Anterior	4.18 ± 0.85	935.43 ± 160.94	3.55 ± 0.75	805.92 ± 167.40
Lateral	5.55 ± 1.07	1146.32 ± 151.33	4.72 ± 0.91	1079.23 ± 157.23
Posterior	3.50 ± 0.83	653.37 ± 157.43	2.83 ± 0.79	504.13 ± 130.84
*F*	239.63	290.11	512.52	756.47
*p*	0.000	0.000	0.000	0.000
Plane 8				
Medial	6.55 ± 1.40	1175.90 ± 177.96	5.86 ± 1.17	1142.28 ± 154.01
Anterior	4.77 ± 0.80	1013.86 ± 155.37	4.07 ± 0.82	893.43 ± 167.68
Lateral	6.35 ± 1.15	1233.44 ± 150.26	5.47 ± 1.03	1167.00 ± 143.20
Posterior	4.25 ± 0.98	790.71 ± 180.50	3.51 ± 1.44	653.37 ± 175.61
*F*	144.48	191.39	231.10	541.09
*p*	0.000	0.000	0.000	0.000

Abbreviations: CM, cortical mean density; CTh, cortical thickness.

**TABLE 4:** Comparison of CTh and CM values (Mean ± SD, mm, HU) of the femoral trochanteric region in different ages

Site	Group A1		Group A2		Group A3	
	CTh	CM	CTh	CM	CTh	CM
Plane 4						
Anterior	2.81 ± 0.83	508.66 ± 122.55	2.48 ± 0.69	440.61 ± 133.88	2.33 ± 0.61	410.66 ± 127.16
Lateral	3.74 ± 0.90	519.16 ± 154.66	3.38 ± 0.94	448.02 ± 117.33	3.10 ± 0.90	422.32 ± 124.02
Posterior	1.83 ± 0.42	291.97 ± 66.23	1.83 ± 0.38	269.05 ± 84.98	1.86 ± 0.40	261.87 ± 82.80
*F*	36.33	25.06	206.45	135.18	161.55	113.77
*p*	0.000	0.000	0.000	0.000	0.000	0.000
Plane 5						
Medial	5.78 ± 1.50	1005.71 ± 171.71	5.91 ± 1.40	1056.44 ± 162.63	5.68 ± 1.30	1047.45 ± 166.99
Anterior	3.34 ± 0.82	696.86 ± 176.59	3.12 ± 0.71	660.68 ± 137.10	3.04 ± 0.75	621.44 ± 124.27
Lateral	3.78 ± 0.64	728.98 ± 178.22	3.40 ± 0.78	696.31 ± 153.42	3.34 ± 1.15	644.15 ± 155.39
Posterior	2.29 ± 0.52	447.97 ± 124.04	2.17 ± 0.50	379.03 ± 97.74	2.06 ± 0.48	352.27 ± 96.50
*F*	52.39	42.51	522.99	674.13	450.51	776.63
*p*	0.000	0.000	0.000	0.000	0.000	0.000
Plane 6						
Medial	7.08 ± 2.03	1055.40 ± 172.45	7.17 ± 1.72	1046.68 ± 168.48	6.59 ± 1.71	1027.62 ± 163.59
Anterior	3.41 ± 0.81	777.58 ± 162.22	3.34 ± 0.80	744.38 ± 159.77	3.15 ± 0.73	698.30 ± 156.63
Lateral	4.50 ± 0.80	928.64 ± 165.20	4.29 ± 0.95	945.95 ± 160.39	4.06 ± 1.02	893.14 ± 179.90
Posterior	3.02 ± 0.59	550.69 ± 125.79	2.63 ± 0.71	461.49 ± 115.27	2.42 ± 0.60	443.45 ± 122.52
*F*	51.04	41.79	544.70	490.33	493.83	474.37
*p*	0.000	0.000	0.000	0.000	0.000	0.000
Plane 7						
Medial	7.09 ± 2.30	1091.02 ± 147.43	6.65 ± 1.52	1101.71 ± 152.59	6.25 ± 1.53	1094.12 ± 167.36
Anterior	4.24 ± 0.77	914.65 ± 138.42	3.86 ± 0.85	872.81 ± 171.57	3.65 ± 0.81	826.00 ± 180.97
Lateral	5.42 ± 0.88	1088.92 ± 163.31	5.16 ± 1.01	1113.64 ± 152.56	4.84 ± 1.06	1095.50 ± 163.11
Posterior	3.66 ± 0.62	717.55 ± 167.22	3.17 ± 0.94	572.14 ± 149.12	2.90 ± 0.78	525.13 ± 151.95
*F*	28.58	29.00	327.79	449.48	328.03	485.08
*p*	0.000	0.000	0.000	0.000	0.000	0.000
Plane 8						
Medial	6.76 ± 1.64	1156.22 ± 133.66	6.27 ± 1.25	1153.07 ± 175.10	5.88 ± 1.27	1156.32 ± 156.24
Anterior	4.90 ± 0.84	1001.28 ± 155.63	4.37 ± 0.89	957.82 ± 169.02	4.21 ± 0.85	909.22 ± 174.90
Lateral	6.12 ± 0.91	1162.03 ± 160.50	5.95 ± 1.17	1204.30 ± 138.85	5.59 ± 1.13	1181.84 ± 156.36
Posterior	4.31 ± 0.69	825.97 ± 165.84	3.91 ± 1.02	724.16 ± 194.36	3.59 ± 1.61	667.87 ± 177.97
*F*	23.35	23.27	193.99	279.70	141.06	380.10
*p*	0.000	0.000	0.000	0.000	0.000	0.000

Abbreviations: CM, cortical mean density; CTh, cortical thickness.

**TABLE 5:** Comparison of ULL values (Mean ± SD, mm) in different sexes and ages

Site	Group G1	Group G2	Group A1	Group A2	Group A3
ULL value in Plane 1	25.07 ± 4.24	19.87 ± 3.25	23.92 ± 4.15	21.71 ± 4.59	21.51 ± 4.21
ULL value in Plane 2	29.50 ± 3.59	24.10 ± 3.16	28.79 ± 4.54	26.13 ± 4.31	25.63 ± 3.96
ULL value in Plane 3	39.10 ± 4.92	33.49 ± 4.87	37.41 ± 6.28	35.71 ± 5.79	35.09 ± 5.24
*F*	378.93	790.87	39.85	359.10	435.34
*p*	0.000	0.000	0.000	0.000	0.000

Abbreviation: ULL, upper‐lower diameter length.

**TABLE 6:** Comparison of MLL values (Mean ± SD, mm) in different sexes and ages

Site	Group G1	Group G2	Group A1	Group A2	Group A3
MLL value in Plane 4	53.07 ± 3.95	46.92 ± 3.27	51.45 ± 5.66	49.30 ± 4.35	48.70 ± 4.62
MLL value in Plane 5	37.61 ± 4.48	34.07 ± 3.65	36.82 ± 4.79	35.21 ± 4.16	35.30 ± 4.39
MLL value in Plane 6	28.33 ± 3.41	26.12 ± 3.07	27.97 ± 3.75	26.69 ± 3.13	26.99 ± 3.52
MLL value in Plane 7	23.17 ± 2.87	21.81 ± 2.37	22.25 ± 2.86	22.26 ± 2.44	22.34 ± 2.80
MLL value in Plane 8	20.66 ± 2.74	19.22 ± 2.30	19.54 ± 3.22	19.73 ± 2.40	19.76 ± 2.62
*F*	1857.84	3387.53	209.23	2124.30	1843.13
*p*	0.000	0.000	0.000	0.000	0.000

Abbreviation: MLL, medial‐lateral diameter length.

**TABLE 7:** Maximum von Mises stress value (MPa) of each wall in Planes 1–8 of Models 1–6

Site	Model 1	Model 2	Model 3	Model 4	Model 5	Model 6
Plane 1						
Upper wall	19.29	16.87	32.20	24.60	43.46	39.17
Anterior wall	5.83	8.82	8.87	9.44	17.48	14.10
Lower wall	64.02	54.99	100.76	44.72	84.26	133.26
Posterior wall	2.34	2.85	22.55	5.25	8.75	6.57
Plane 2						
Upper wall	39.51	48.91	26.45	55.58	32.09	59.61
Anterior wall	4.13	7.36	13.16	18.88	16.33	9.21
Lower wall	111.58	87.01	109.13	95.85	133.67	134.71
Posterior wall	9.84	9.45	24.02	18.22	14	14.67
Plane 3						
Upper wall	20.24	6.18	28.82	27.73	45.41	39.82
Anterior wall	12.06	8.05	10.20	15.93	8.65	15.70
Lower wall	118.72	58.80	98.60	85.80	140.74	94.50
Posterior wall	13.77	14.81	28.51	22.79	31.72	27.03
Plane 4						
Anterior wall	8.60	7.37	9.06	12.43	18.39	9.23
Lateral wall	6.87	4.53	9.68	5.51	21.65	9.74
Posterior wall	6.43	4.07	7.99	8.23	12.01	6.18
Plane 5						
Medial wall	79.20	32.22	82.98	52.66	90.03	74.24
Anterior wall	20.36	20.75	29.94	25.18	37.46	31.52
Lateral wall	24.66	20.51	50.52	34.36	77.67	41.79
Posterior wall	6.24	4.52	10.36	15.02	19.43	7.68
Plane 6						
Medial wall	72.12	46.96	104.19	51.35	101.69	66.80
Anterior wall	22.56	21.49	32.77	23.14	46.16	33.70
Lateral wall	46.74	38.22	48.22	43.07	85.47	56.28
Posterior wall	7.25	6.80	17.42	13.28	12.41	12.54
Plane 7						
Medial wall	81.22	55.52	103.94	52.40	139.96	73.01
Anterior wall	20.42	13.23	31.57	16.05	30.50	24.52
Lateral wall	48.60	41.67	88.06	53.19	72.19	69.28
Posterior wall	11.47	6.82	15.26	11.81	21.20	16.22
Plane 8						
Medial wall	75.15	55.89	105.60	64.57	106.89	61.68
Anterior wall	21.41	12.38	27.31	9.75	71.09	25.58
Lateral wall	46.91	37.96	87.66	54.53	73.23	61.68
Posterior wall	14.38	4.44	6.72	19.80	57.58	11.40

### Finite Element Analysis of the Proximal Femur

**Fig. 6:**
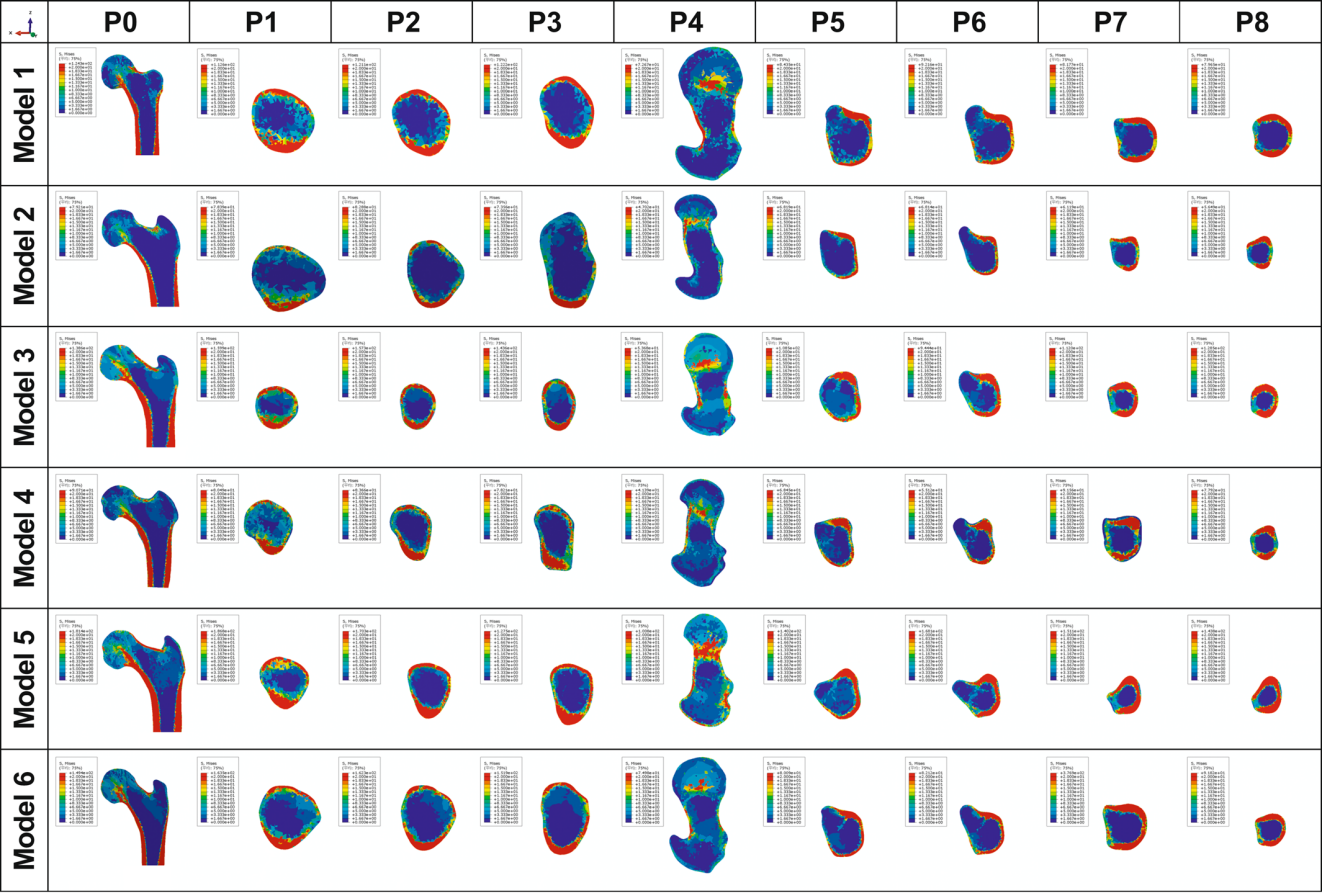
The von Mises stress distribution of Models 1–6. (P0): The von Mises stress distribution in the coronal plane of the proximal femur. (P1–3): The von Mises stress distribution of the femoral neck region. P1, plane at the subcephalic of the femoral neck; P2, plane at the middle of the femoral neck; P3, plane at the bottom of the femoral neck, respectively. (P4–8): The von Mises stress distribution of the trochanteric region. P4, plane at the 20mm above the upper edge of femoral lesser trochanter; P5, plane at the upper edge of femoral lesser trochanter; P6, plane at the vertex of femoral lesser trochanter; P7, plane at the lower edge of femoral lesser trochanter; P8, plane at the 20mm below the vertex of femoral lesser trochanter

In the simulation of models 1–6, the von Mises stress was concentrated on the upper and lower walls in planes 1–3 and concentrated on the medial and lateral walls in planes 5–8 (Figure 6).

**Fig. 7:**
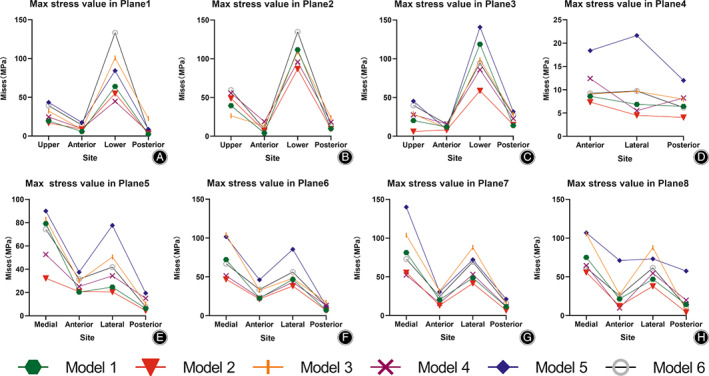
Maximum von Mises stress values of each wall in planes 1–8 of Models 1–6

In the femoral neck, the maximum von Mises stress values of the upper and lower walls were higher than those of the anterior and posterior walls in planes 1–3 (Figure 7A–C and Table 7). In the femoral trochanteric region, the maximum von Mises stress values of the medial and lateral walls were higher than those of the anterior and posterior walls in planes 5–8 (Figure 7E–H and Table 7).

The number of elements in models 1–6 is shown in Supplementary Table 1.

### Intraobserver and Interobserver Reliability

The ICC showed values of >0.80 for interobserver and intraobserver reliability (Supplementary Table 2).

## Discussion

In this study, we verified the high‐modulus mechanical structure of the proximal femur. Our major findings were as follows: (1) the high‐modulus structure of cortical bone varied dramatically across cross‐sections independent of age and sex, with prominent cortical thickening, densification, and stress concentrating at the upper, medial, and lateral walls; (2) the above three walls converged to a triangle in the coronal plane, independent of age and sex.

### The High‐Modulus Structural Morphology of Bone Mass in the Proximal Femur

The proximal femur consists of cancellous and cortical bone, bearing 15%–53% and 47%–85% of the stress load, respectively^20^, ^21^. However, the previous understanding of the mechanical structure was mainly based on the morphology of the cancellous bone^31^, without investigation of different sex and age groups. In this study, the anatomical parameters of the proximal femoral cortical bone in patients of different ages and genders were measured, and heterogeneity of the cortical bone structure was found. In the femoral neck region, the CTh and CM values of the upper and lower walls were higher than those of the anterior and posterior walls, regardless of sex and age. The only exception was that the upper walls exhibited the lowest CM value in plane 3, which might be caused by the anatomical structure of the junction between the upper wall and greater trochanter, where the principal tensile trabecula is separated from the upper cortical bone^32^. In the trochanteric region, the medial and lateral walls had higher CTh and CM values than those of the anterior and posterior walls, regardless of sex and age.

Previous studies have demonstrated that the cortical geometry and microarchitecture of the proximal femoral structure display regional heterogeneity. Khoo^33^ found that the mineral masses of the upper and lower segments were larger than those of the anterior and posterior femoral necks. Through a study of the histomorphometric properties of the subtrochanteric femoral region, Tong^34^ found that the cortical widths in the medial and lateral quadrants were significantly higher than those detected in the anterior and posterior quadrants. However, the cortical bone density was not measured in these studies.

According to Wolff's law^35^, ^36^, local bone tissue normally adapts its density and structure to the current loads, so it can be inferred that the medial, lateral, and upper walls bear the main stress of the proximal femur regardless of sex and age. Concurrently, we found that ULL values increased from the inside to the outside, and the MLL values decreased gradually from top to bottom regardless of sex and age; therefore, it can be inferred that the medial, lateral, and upper walls of the proximal femur converge in a triangular structure in the coronal plane and bear the main stress.

### The Mechanical Effect of Triangular High‐Modulus Structure in the Proximal Femur

The finite element analysis in this study found that the main stress in the proximal femur was concentrated in the upper, medial, and lateral walls, which confirmed that the triangular structure bears the main stress in the proximal femur. Through finite element analysis of the femoral neck, Nawathe^20^ found that stress was mainly concentrated in the upper and lower lateral walls. Endo^37^ proposed that stress was mainly concentrated in the medial and lateral walls using finite element analysis of the femoral shaft. However, the above studies were based on the mechanical verification of individuals and did not consider the proximal femur as a whole for mechanical testing, nor did they investigate the mechanical structure of the bone cortex.

**Fig. 8:**
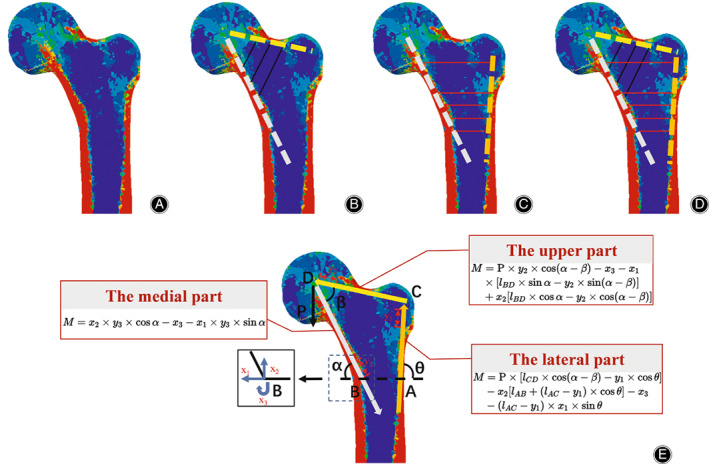
Mechanical stress nephogram: (A) Stress distribution of proximal femur. (B) The stress on the upper and lower walls of the femoral neck displays a convergent trend. (C) The medial and lateral stress of the trochanteric region shows a convergent tendency. (D) Mechanical stress shows a triangular structure. (E) Quantitative calculation of the relationship between triangular morphological structure and mechanical function of the proximal femur

According to the stress distribution law of the proximal femur, the upper, medial, and lateral edges of the proximal femoral coronal plane are defined as follows: (1) the upper edge is the high‐stress area of bone along the upper part of the femoral neck, which runs from the circle point of the femoral head to the cortex of the vastus lateralis ridge; (2) the medial edge is the high‐stress area of bone extending from the femoral head, along the medial cortex to the distal end; and (3) the lateral edge runs from the vastus lateralis ridge along the high‐stress area of the lateral wall to the distal end. At the same sites, the cortical bone fusing with principal compressive, principal tensile, secondary compressive, secondary tensile, and greater trochanter trabeculae forms a triangular mechanical structure to maintain mechanical stability (Figure 8). Meanwhile, as the junction of the upper and lateral walls, the greater trochanter realizes the effective transmission of tensile stress, which has not been mentioned in previous literature, to the best of our knowledge.

The structure has obvious mechanical advantages: (1) the medial part forms the oblique support of the proximal femoral cantilever structure, greatly reducing the bending stress and deflection of the structure; (2) the upper part acts as a connection between the medial and lateral edges of the proximal femur, resisting the bending moment caused by physiological loads; and (3) the lateral part can effectively reduce the sliding and deflection of the femoral neck under physiological loads. Under a physiological load, the complete mechanical triangular structure can effectively reduce the bending moment, balance the shear force of the physiological load, realize a balanced distribution of stress in the structure, and maintain balance and stability. Therefore, we propose a theory for triangular hip stabilization reconstruction. We believe that the treatment principle of hip fracture is to reconstruct the overall structure to achieve a low bending moment and self‐balanced stress distribution bone on the fixation and skeletal system, and further obtain firm mechanical stability for patients to walk. During the operation, if any part of the triangular structure is not well reconstructed, it cannot form a strong mechanical stability and will lead to the collapse of the structure, resulting in treatment failure.

When fractures occur, the integrity of the mechanical structure is destroyed, which leads to mechanical instability. Complete reconstruction of the triangular mechanical structure is key to a successful operation. Any technical means to achieve the overall reconstruction of the triangular mechanical structure can achieve mechanical stability in this region. Ye *et al*.^38^ used a hollow screw combined with a medial support plate to treat patients with femoral neck fractures, and all patients healed without any postoperative complications. Through biomechanical experiments, Kunapuli^39^ confirmed that the overall reconstruction of the three parts of the proximal femur had a significant advantage in terms of mechanical stability. Based on this mechanical structure, we designed a series of fixation instruments that have been used in the treatment of hip fractures with satisfactory clinical results^18^, ^40^. Both theory and practice have proven the reliability of a triangular mechanical structure.

### Strengths and Limitations

Through the combination of anatomical measurement and finite element analysis, our study confirmed the existence of a triangular mechanical structure of the proximal femur from the perspective of morphology and function and provided a new perspective for the exploration of the mechanical structure of human bones. This study had some limitations. First, the sample size of some age groups was limited. The inclusion of additional groups could strengthen the representation in the study. Second, the subjects in this study were patients with femoral intertrochanteric fractures. A normal population should be included to ascertain the universality of the results.

### Conclusions

In this study, we identified a triangular structure of bone mass concentration in the proximal femur using anatomical measurements and further confirmed that this structure bears the main stress transmission in the proximal femur through finite element analysis. The triangular mechanical structure proposed in this study will help guide future improvements in the surgical strategy and instrument design of the proximal femur.

## Author Contributions

Conception: PF Tang, LC Zhang, and H Zhang; Collection, measurement, and analysis of data: GX Xu, JT Li, H Li, and DF Wang; Mechanical analysis: C Xu and D Xiong; Preparation of the manuscript: GX Xu, JT Li, and C Xu; Revision for important intellectual content: GX Xu, JT Li, and PF Tang; Supervision: PF Tang, LC Zhang, and H Zhang.

## Etics Statement

The ethics statement has been provided in Methods section: This study was approved by the institutional review board of our hospital (S2020‐114‐04).

## Supporting information


**Supplementary Table 1** Number of elements and nodes in the Models 1–6.
**Supplementary Table 2** Reliability study results.
