# Supplementary material for: Triangular Mechanical Structure of the Proximal Femur
Source: Orthop Surg. 2022 Sep 30;14(11):3047–60. doi: 10.1111/os.13498 (PMC9627078; doi:10.1111/os.13498)
Supplement: Supplementary file 1 — Supplementary Table 1 Number of elements and nodes in the Models 1–6. Supplementary Table 2 Reliability study results. [file OS-14-3047-s001.docx]

**Supplementary Table 1.** Number of elements and nodes in the Models 1-6.

|  | Model 1 | Model 2 | Model 3 | Model 4 | Model 5 | Model 6 |
| --- | --- | --- | --- | --- | --- | --- |
| Elements | 124776 | 103676 | 102192 | 145197 | 216663 | 274748 |
| Nodes | 24677 | 20644 | 20278 | 28440 | 41582 | 52704 |

**Supplementary Table 2.** Reliability study results

| Items | Intraobserver | | Interobserver | |
| --- | --- | --- | --- | --- |
|  | ICC | 95% CI | ICC | 95% CI |
| CTh (CM) value in Plane1 |  |  |  |  |
| Upper | 0.864 (0.871) | 0.725-0.940 (0.754-0.942) | 0.856 (0.867) | 0.694-0.938 (0.747-0.940) |
| Anterior | 0.827 (0.835) | 0.679-0.920 (0.689-0.925) | 0.811 (0.803) | 0.653-0.913 (0.637-0.909) |
| Lower | 0.970 (0.845) | 0.916-0.989 (0.706-0.930) | 0.965 (0.841) | 0.897-0.987 (0.699-0.928) |
| Posterior | 0.868 (0.846) | 0.748-0.940 (0.711-0.930) | 0.831 (0.843) | 0.685-0.923 (0.706-0.928) |
| CTh (CM) value in Plane2 |  |  |  |  |
| Upper | 0.831 (0.822) | 0.686-0.923 (0.669-0.919) | 0.824 (0.814) | 0.675-0.919 (0.656-0.915) |
| Anterior | 0.822 (0.885) | 0.668-0.918 (0.778-0.949) | 0.813 (0.878) | 0.657-0.914 (0.753-0.946) |
| Lower | 0.904 (0.817) | 0.812-0.957 (0.609-0.922) | 0.858 (0.813) | 0.727-0.936 (0.609-0.919) |
| Posterior | 0.814 (0.818) | 0.642-0.916 (0.665-0.916) | 0.803 (0.812) | 0.637-0.909 (0.655-0.913) |
| CTh (CM) value in Plane3 |  |  |  |  |
| Upper | 0.851 (0.839) | 0.719-0.932 (0.698-0.926) | 0.844 (0.838) | 0.706-0.929 (0.697-0.926) |
| Anterior | 0.848 (0.872) | 0.712-0.931 (0.755-0.942) | 0.827 (0.866) | 0.678-0.921 (0.745-0.939) |
| Lower | 0.870 (0.840) | 0.750-0.941 (0.701-0.927) | 0.859 (0.839) | 0.730-0.936 (0.699-0.927) |
| Posterior | 0.825 (0.855) | 0.89-0.978 (0.726-0.934) | 0.802 (0.822) | 0.636-0.909 (0.670-0.918) |
| CTh (CM) value in Plane4 |  |  |  |  |
| Anterior | 0.847 (0.873) | 0.650-0.937 (0.755-0.943) | 0.803 (0.870) | 0.598-0.914 (0.751-0.942) |
| Lateral | 0.843 (0.868) | 0.693-0.930 (0.748-0.940) | 0.837 (0.867) | 0.685-0.927 (0.747-0.940) |
| Posterior | 0.835 (0.843) | 0.692-0.924 (0.693-0.930) | 0.802 (0.898) | 0.639-0.908 (0.801-0.955) |
| CTh (CM) value in Plane5 |  |  |  |  |
| Medial | 0.856 (0.858) | 0.708-0.937 (0.732-0.936) | 0.836 (0.850) | 0.687-0.926 (0.715-0.932) |
| Anterior | 0.846 (0.931) | 0.671-0.934 (0.855-0.970) | 0.812 (0.929) | 0.638-0.915 (0.849-0.969) |
| Lateral | 0.842 (0.938) | 0.704-0.928 (0.876-0.973) | 0.828 (0.936) | 0.681-0.921 (0.873-0.972) |
| Posterior | 0.823 (0.902) | 0.671-0.919 (0.808-0.956) | 0.805 (0.817) | 0.640-0.910 (0.660-0.916) |
| CTh (CM) value in Plane6 |  |  |  |  |
| Medial | 0.890 (0.883) | 0.775-0.952 (0.774-0.947) | 0.880 (0.879) | 0.757-0.948 (0.768-0.946) |
| Anterior | 0.921 (0.854) | 0.842-0.965 (0.725-0.934) | 0.902 (0.849) | 0.808-0.956 (0.716-0.931) |
| Lateral | 0.852 (0.876) | 0.708-0.935 (0.762-0.945) | 0.843 (0.867) | 0.700-0.929 (0.745-0.940) |
| Posterior | 0.832 (0.873) | 0.687-0.923 (0.757-0.943) | 0.815 (0.869) | 0.657-0.915 (0.750-0.941) |
| CTh (CM) value in Plane7 |  |  |  |  |
| Medial | 0.901 (0.832) | 0.652-0.914 (0.681-0.924) | 0.900 (0.802) | 0.786-0.957 (0.633-0.909) |
| Anterior | 0.862 (0.963) | 0.637-0.947 (0.923-0.984) | 0.867 (0.962) | 0.677-0.947 (0.922-0.983) |
| Lateral | 0.894 (0.925) | 0.783-0.954 (0.844-0.968) | 0.892 (0.922) | 0.788-0.952 (0.831-0.967) |
| Posterior | 0.888 (0.960) | 0.784-0.950 (0.918-0.983) | 0.872 (0.959) | 0.755-0.943 (0.917-0.982) |
| CTh (CM) value in Plane8 |  |  |  |  |
| Medial | 0.845 (0.842) | 0.706-0.930 (0.699-0.929) | 0.867 (0.839) | 0.745-0.940 (0.691-0.927) |
| Anterior | 0.863 (0.891) | 0.736-0.938 (0.763-0.954) | 0.866 (0.893) | 0.740-0.940 (0.770-0.955) |
| Lateral | 0.873 (0.862) | 0.722-0.947 (0.719-0.940) | 0.879 (0.860) | 0.736-0.949 (0.711-0.939) |
| Posterior | 0.849 (0.864) | 0.715-0.931 (0.737-0.939) | 0.839 (0.866) | 0.700-0.927 (0.742-0.940) |
| ULL in Plane1 | 0.869 | 0.750-0.941 | 0.814 | 0.657-0.914 |
| ULL in Plane2 | 0.871 | 0.473-0.958 | 0.862 | 0.495-0.954 |
| ULL in Plane3 | 0.817 | 0.566-0.926 | 0.814 | 0.572-0.924 |
| MLL in Plane4 | 0.949 | 0.897-0.978 | 0.910 | 0.823-0.960 |
| MLL in Plane5 | 0.889 | 0.784-0.950 | 0.846 | 0.710-0.930 |
| MLL in Plane6 | 0.946 | 0.890-0.977 | 0.888 | 0.784-0.950 |
| MLL in Plane7 | 0.943 | 0.886-0.975 | 0.854 | 0.724-0.934 |
| MLL in Plane8 | 0.816 | 0.655-0.916 | 0.850 | 0.717-0.932 |

CTh=cortical thickness, CM=cortical mean density, ULL=upper-lower diameter length, MLL=medial-lateral diameter length
